# Hospital work environments affect the patient safety climate: A longitudinal follow-up using a logistic regression analysis model

**DOI:** 10.1371/journal.pone.0258471

**Published:** 2021-10-15

**Authors:** Kirsten Brubakk, Martin Veel Svendsen, Ellen Tveter Deilkås, Dag Hofoss, Paul Barach, Ole Tjomsland

**Affiliations:** 1 Department of Human Resources, South Eastern Norway Regional Health Authority, Hamar, Norway; 2 Institute for Health and Society, University of Oslo, Oslo, Norway; 3 Department of Occupational and Environmental Medicine, Telemark Hospital Trust, Skien, Norway; 4 Department of Quality Improvement and Patient Safety, Norwegian Directorate of Health, Oslo, Norway; 5 Unit for Health Services Research, Akershus University Hospital, Lørenskog, Norway; 6 Department of Health and Inequality, National Institute of Public Health, Oslo, Norway; 7 Department of Pediatrics, Wayne State University, Detroit, Michigan, United States of America; 8 Jefferson College of Population Health, Philadelphia, Pennsylvania, United States of America; 9 Sigmund Freud University, Vienna, Austria; 10 Department of Health, South Eastern Norway Regional Health Authority, Hamar, Norway; University of Hradec Kralove: Univerzita Hradec Kralove, CZECH REPUBLIC

## Abstract

**Background:**

Occupational worker wellness and safety climate are key determinants of healthcare organizations’ ability to reduce medical harm to patients while supporting their employees. We designed a longitudinal study to evaluate the association between work environment characteristics and the patient safety climate in hospital units.

**Methods:**

Primary data were collected from Norwegian hospital staff from 970 clinical units in all 21 hospitals of the South-Eastern Norway Health Region using the validated Norwegian Work Environment Survey and the Norwegian version of the Safety Attitudes Questionnaire. Responses from 91,225 surveys were collected over a three year period. We calculated the factor mean score and a binary outcome to measure study outcomes. The relationship between the hospital unit characteristics and the observed changes in the safety climate was analyzed by linear and logistic regression models.

**Results:**

A work environment conducive to safe incident reporting, innovation, and teamwork was found to be significant for positive changes in the safety climate. In addition, a work environment supportive of patient needs and staff commitment to their workplace was significant for maintaining a mature safety climate over time.

**Conclusions:**

A supportive work environment is essential for patient safety. The characteristics of the hospital units were significantly associated with the unit’s safety climate scores, hence improvements in working conditions are needed for enhancing patient safety.

## Introduction

Providing high value, patient-centered, and quality care while preventing patient harm remains a worldwide challenge [1]. During the past two decades, acute-care hospitals have been challenged as never before to develop and sustain operating systems to ensure patient safety. Many factors, latent and active, interact to cause adverse events [2] and Vincent and colleagues describe safety climate and work environment as important factors influencing clinical practice [3]. Healthcare organizations must consider issues across whole systems, including organizational and cultural factors affecting the system in which care is provided if they are to improve their patient outcomes [4,5].

Patient safety culture, a specific aspect of organizational culture, is increasingly recognized as a critical determinant in reducing patient risk due to adverse medical care [1,4,6,7]. Patient safety culture refers to the collection of individual and group values, attitudes, and practices that guide hospital staff behavior [8]. Addressing organizational culture is viewed as essential to health system transformation [9,10] and remains an important factor in the successful implementation and sustainability of quality improvement initiatives on the front lines of care [11]. The organization’s culture also shapes staff perceptions of “normal” behavior. In essence, the culture on the front line of care is “the way things are done here” and is highly influenced by the organization-wide culture and norms [12]. Zhou et al. captured this well, saying that *“the safety culture of an organization can motivate workers to engage in safe behaviors and facilitate the translation of these behaviors into daily practice*, *and can also influence the ability of staff to raise concerns regarding safety and the ability of managers to respond to those concerns”* [13].

According to most up to date safety science, the analysis of working processes and organizational conditions are necessary to understand how adverse events can be prevented [14,15]. There is significant potential to enhance patient safety performance and eliminate hazards in work environments with a mature patient safety culture [16,17]. The staff perceptions of their work environment can vary over time with changes in work and the psycho-social working conditions including leadership, patient safety climate, competence, training, ability to safety speak up, and organizational design characteristics [18–21]. These factors may influence safety precautions, routines, and ultimately patient safety and quality of care. Organizations with diverging cultural perceptions and low social trust among staff are regarded as having weak and immature cultures, with a limited ability to nurture and support staff best practices, and often leading to unpredictable and harmful outcomes [22]. A consistent association between a positive (mature) patient safety culture and beneficial clinical outcomes is demonstrated in previous studies [20,23–27]. Safety culture is necessary to shape front-line staff behaviors and encourage safe-conduct [28]. Reliably measuring patient safety culture is challenging [29]. A promising approach to assess the safety culture in caregiving units is to use validated questionnaires [30]. According to Sexton et al., when using questionnaires to study group-level perceptions, the most appropriate term to use is climate [31]. Climate refers to the shared perceptions about norms, processes, and policies related to patient safety and provides a snapshot of how staff perceive aspects of their culture [30].

We do not fully understand what factors explain the wide variation in culture despite the emphasis on safety culture as an important strategy to patient safety [4,12,32,33]. We hypothesize that the work environment is related to how patient safety is handled on care giving unit. This study aims to explore the association between work environment characteristics and the development in safety climate.

## Materials and methods

### Design and data sources

This study was conducted using a longitudinal prospective design, combining data from the validated annual Work Environment Survey (WES) and the safety climate data from the Norwegian Safety Attitude Questionnaire (SAQ), both country-wide, large multisite organizational surveys.

### Setting and sample

Hospital staff with more than three months, or 30% employment before the survey administration at 21 hospitals in nine hospital trusts in South-Eastern Norway were eligible for inclusion. Two of the hospitals were teaching hospitals with > 600 beds, 6 hospitals had < 100 beds, and one hospital was a rehabilitation hospital. The sample for this study was retrieved from the 970 clinical units participating in all three surveys (WES 2011, SAQ 2012 and SAQ 2014) with more than five responders from each unit and where no major reorganization had taken place between 2011 and 2014. Clinical units were defined as units where employees have direct patient contact.

### Questionnaire

Two survey instruments provided data for this study. The Norwegian SAQ, adapted from the Safety Attitude Questionnaire, generic version (SAQ) [34,35] and validated in Norwegian settings [36] was used to evaluate the safety climate among staff. The Work Environment Survey (WES), based on the General Nordic Questionnaire for Psychological and Social Factors at Work (QPSnordic) [37] was used to evaluate staff perceptions about their work environment characteristics.

#### Safety Attitude Questionnaire (SAQ) (Table 1)

**Table 1:** Safety Attitudes Questionnaire (SAQ) factors and items.

FACTOR	Items
Safety Climate	I would feel safe being treated here as a patient Medical errors are handled appropriately in this unit I know the proper channels to direct questions regarding patient safety in this unit I receive appropriate feedback about my performance In this unit, it is difficult to discuss errors I am encouraged by my colleagues to report any patient safety concerns I may have The culture in this unit makes it easy to learn from the errors of others

The Norwegian SAQ used for the National Patient Safety Campaign consists of the factors Teamwork Climate and Safety Climate [38]. However, for this study only data retrieved from the safety climate factor were included. The exclusion of a factor was done to minimize the overlap of items between the WES and SAQ surveys. The subset of safety climate from the larger SAQ has previously been validated and the psychometrics are sound [12]. The safety climate factor consists of seven unit level items presented in Table 1, addressing staff perspectives concerning patient safety, support and feedback, and incident reporting. All items were scored on a five-point Likert scale (i.e., from “1 = strongly disagree”, “2 = disagree”, “3 = neutral”, “4 = agree” and “5 = strongly agree”) and were converted to a 0–100 scale [39] and given the values 0, 25, 50, 75, 100. A score of zero represents the most undesirable result, and 100 represents the most desirable. Negatively worded items were reversely scored to match positively worded items.

We ascribed a mature safety climate to units where more than 60% of the staff responded positively to the safety climate items (scores above 75 on a 0–100 point scale). The Norwegian Directorate of Health used this definition in its national report on patient safety culture measurements in 2012 and 2014 [40]. The definition is based partly on Pronovost et al. in their assessment of progress toward improving safety culture by achieving at least 60% agreement at the unit-level and in line with Zohar et al. who defined climate strength by the degree of staff consensus about the importance of patient safety [22,41].

#### The Work Environment Survey (WES) (Table 2)

**Table 2:** Work Environment Survey (WES) factors and items.

Factors	Items
Improvement	In my unit, we do well in reporting and follow up on adverse events It is safe to report adverse events in my/this unit We openly discuss adverse events and learn from them In this unit, we encourage each other to think of ways to do things better
Quality	In my unit different professions collaborate well We work efficiently in my unit In my unit high quality is maintained
Patient-Centered	In my unit, we listen to the views of patients/clients In my unit, we are available to patients/clients In my unit, sufficient information is given to patients/clients
Respect	In my unit, we respect patients’/clients’ cultural background and religion In my unit, we ensure that we keep made appointments In my unit, we communicate clearly and in an understandable way
Motivation	Is your work challenging in a positive way My work tasks motivate me The work is so interesting in itself that it is strongly motivating
Engagement	Do you look forward to go to work How often does dissatisfaction with your work make you want to change employer Overall, how satisfied are you with the work you do now
Commitment	To my friends, I praise this organization as a great place to work This organization inspires me to give my very best job performance I am proud of my workplace
Personal Development	I can develop professionally through my work I get sufficient training and advice to do a good job Is your work organized in a way that lets you improve your capacities Do you get feedback about the quality of the work you do
Empowerment	Are you encouraged to participate in decision making Are you encouraged to speak up when you have a different opinion
Role Expectations	Do you know what your responsibilities are Do you know what is expected of you at work
Social Climate	Is the social climate in your unit characterized by a team spirit If needed, can you get support and help from your coworkers Do you perceive good collaboration in your unit
Conflict	Have you observed anyone being harassed or bullied at your workplace during the last six months Have you noticed disruptive conflicts in your unit When conflicts occur, are they handled in a professional manner
Workload	Is the physical load of your work too heavy Is your work pace challenging Is your workload challenging Do you perform work tasks for which you need more training
Autonomy	Can you influence the amount of work assigned to you Can you set your own work pace
Role conflicts	Do you have to perform procedures which you feel should be done differently Are you given assignments without adequate resources to complete them Do you receive incompatible requests
Sick leave	Issues at work have contributed to my sick leaves during the last 12 months
Leadership	My immediate superior is available to me when I need it My immediate superior does an excellent job of giving us information about what goes on in our organization My immediate superior makes clear performance demands My immediate superior adheres to what we have agreed upon If I were subjected to violence or threats, I could count on the support of my immediate superior If I were sick for a more extended period, I could count on the support of my immediate superior
Patient Safety Culture	I would feel safe if I was a patient here Adverse medical events are appropriately handled here

The Work Environment Survey (WES) instrument is a validated work environment questionnaire based on QPSnordic. The questionnaire is adapted to the Nordic context to provide a comprehensive picture of workers’ perceptions about their work environment [37]. The instrument includes 18 factors, with each factor consisting of 1 to 6 items, please see Table 2. The response to each item is rated using a 5-point Likert scale (for some items “1 = Strongly disagree”, “2 = Disagree”, “3 = Neither disagree nor agree”, “4 = Agree”, “5 = Strongly agree” or, where appropriate, “1 = Never/very seldom”, “2 = Seldom”, “3 = Sometimes”, “4 = Quite often”, “5 = Very often/always”) and each item is converted to a 0–100 scale. The Patient Safety Culture factor was excluded from the analysis as safety climate was the outcome variable in this study.

### Data collection

The web surveys were distributed by email to eligible staff. Responding to the survey was encouraged by management and time to complete the survey was made available during work hours. Management reminded staff to respond to the survey. WES data was collected in 2011 and SAQ data were collected in years 2012 and 2014. The surveys were anonymous, and identified only with unit affiliation.

### Ethics approval

The Medical and Health Research Ethics Committee (REC) in the South-Eastern Norway Region approved the study with a waiver of informed consent since all data retrieved from the surveys were anonymous.

### Study outcomes

The primary outcome in the study was patient safety climate. We studied three specific outcomes associated with the development of a safety climate:

1. Change in safety climate score over two years (2012–2014).
2. Raising safety climate to a mature level (>60% of staff scores 75 or higher).
3. Maintaining a mature safety climate over time.

### Statistical analysis

Bivariate regression analyses were performed to identify which of the 17 hypothesized explanatory factors listed in Table 2 were significantly associated with improvements in the safety climate scores and with the odds of achieving and maintaining a mature safety climate. Factors with p-values not exceeding 0.05 were included in the multivariate explanatory model.

A stepwise linear regression model was used to assess the work environment characteristics most significant for predicting a change in safety climate score. A backward regression was performed to identify the most significant factors predicting a change in the unit’s safety climate. A forward logistic regression model was used to calculate the predictor odds ratio (OR) of raising a unit’s safety climate to a mature level (yes/no) and in maintaining a mature safety climate level over time (yes/no).

The models’ fit to the data was assessed by the r^2^_adj_ and the Nagelkerke R-squared [42]. To adjust for the potential for improvement at baseline, the unit SAQ_2012_ score was included in all models, as was the hospital unit size. All reported P values are two-sided. P values equal/lower than 0.05 were considered statistically significant. The 95% confidence intervals are presented for B and ORs. The data were analyzed using SPSS statistical software package for Windows (version 25; IBM Corp, Armonk, NY, USA).

## Results

**Fig 1:**
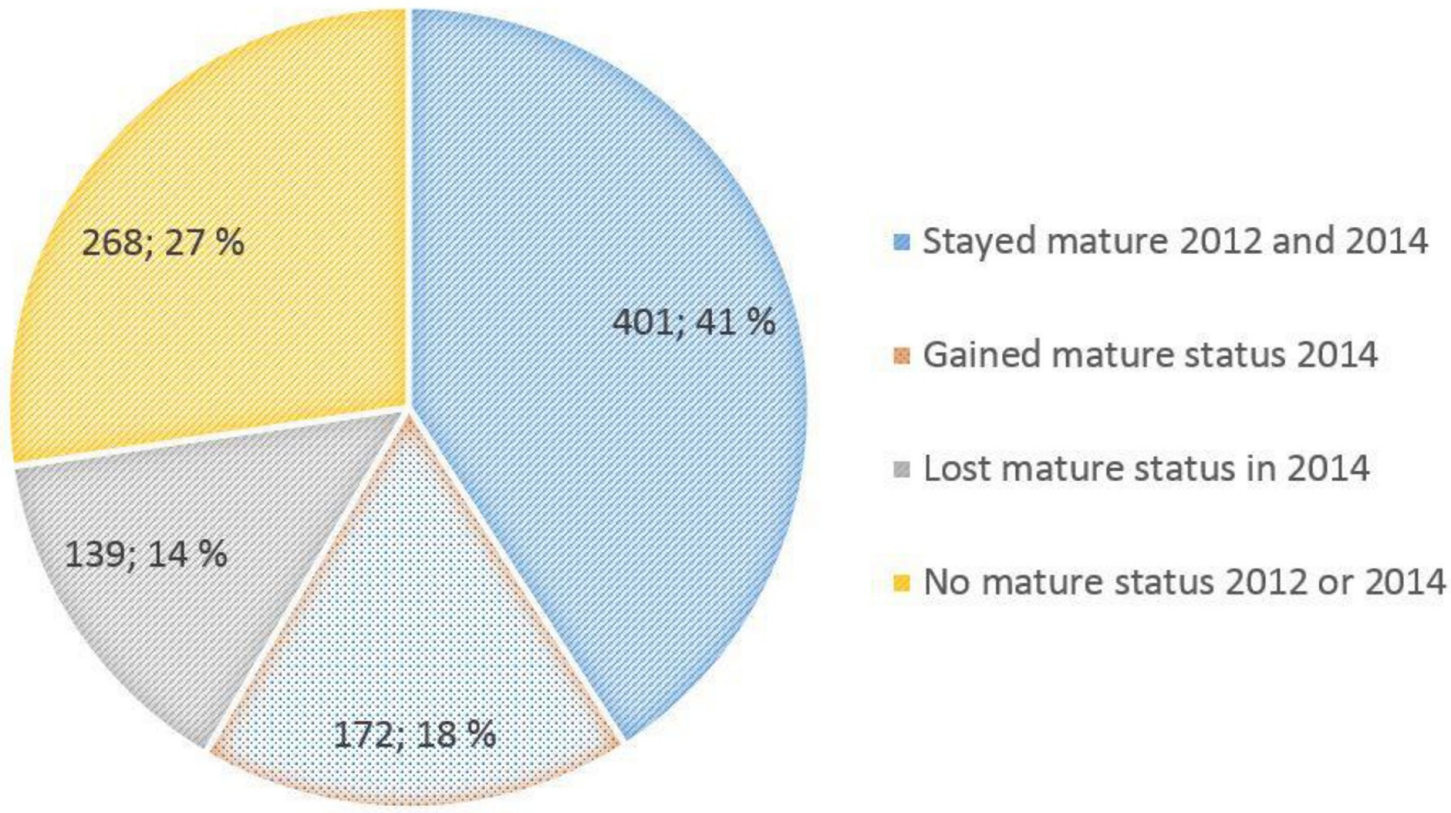
Units that changed their level of mature safety climate between 2012 and 2014, by number of units and percentage.

A total of 91,225 surveys were completed over a three year period. Table 3 shows the response rates ranging from 57% to 77%. The mean size of the included clinical units was 26 employees, ranging from five to 110. Individual perceptions were aggregated by clinical unit, providing a means score (snapshot) of work environment characteristics and safety climate on a given unit [31]. At baseline 2012, 440 units did not have a mature safety climate and were well positioned to improve their safety climate. Five hundred and thirty units had the potential to maintain their mature climate. Fig 1 shows that during the two-year interval studied, 2012–2014, 172 units (18%) raised their safety climate levels to a mature level and 401 units (41%) maintained a level of a mature safety climate.

**Table 3:** Response rate for each survey year.

YEAR	2011 WES	2012 SAQ	2014 SAQ
No. surveys distributed	55 815	40 737	41 052
No. surveys returned	42 977	24 849	23 399
Response rate	77%	61%	57%

Table 4 shows the 14 factors identified by the initial univariate analyses that were included in a multivariate backward regression model adjusted for the SAQ_2012_ and unit size. The data were adjusted for unit size as larger units significantly reported lower WES scores than smaller units and was corroborated by previous research [43]. The variables were eliminated from the regression analysis to identify the model that best explains the data and to reduce the multi-collinearity problems between the factors. Table 5 presents the three factors which significantly predicted a change in the safety climate levels: *Improvement*, *Quality*, *and Patient-Centered*. Together, the three factors explain nearly 30% of the variation found in the hospital unit’s safety climate scores. Change in score is depicted as Δ in the table.

**Table 4:** Each WES factors univariate association with the change in climate score.

Factors	Δ Safety climate score* (n = 970), B(95% CI)
Improvement	**0.15 (0.10, 0.21)**
Quality	**0.18 (0.12, 0.24)**
Patient-Centered	**0.16 (0.10, 0.23)**
Respect	**0.21 (0.14, 0.28)**
Motivation	**0.11 (0.05, 0.16)**
Engagement	**0.09 (0.04, 0.14)**
Commitment	**0.11 (0.06, 0.15)**
Personal Development	**0.08 (0.03, 0.12)**
Empowerment	**0.07 (0.03, 0.11)**
Role Expectation	**0.17 (0.10, 0.24)**
Social Climate	**0.13 (0.07, 0.18)**
Conflict	**0.05 (0.01, 0.09)**
Workload	0.03 (-0.01, 0.07)
Autonomy	0.01 (-0.02, 0.03)
Role Conflict	**0.11 (0.05, 0.17)**
Sick Leave	0.04 (-0.1, 0.09)
Leadership	**0.04 (0.002, 0.09)**

* Adjusted for score SAQ_2012._

Statistical significance at the P < 0.05 level in bold.

**Table 5:** Work environment factors most significantly associated with a change in climate score.

Factors	Δ Safety climate score* (n = 970), B(95% CI)
R^2^_adj_	0.284
Improvement	0.092 (0.030, 0.154)
Quality	0.084 (0.008, 0.161)
Patient-Centered	0.084 (0.009, 0.158)

Only factors significant in at least one of the models are presented.

* Adjusted for unit size and score SAQ_2012._

Statistical significance at the P < 0.05 level.

The logistic regression model analyzed each of the 17 factors adjusted for the SAQ_2012_ and unit size to identify the unit characteristics most significantly associated with development of a unit-level maturity. To raise the safety climate from a non-mature level to a mature level, 12 of the 17 factors needed to be significant at a P<0.05 level (six at the P<0.01). To maintain a mature level, all 17 factors needed to be significant at a P<0.01 level.

The odds ratio (OR) was calculated for the two binary outcome variables: raising safety climate to a mature level (yes/no) and maintaining a mature safety climate level over time (yes/no). Three of the factors were retained in the model: Improvement, Patient-Centeredness, and Commitment (Table 6). Scoring one point higher on the Improvement factors was associated with an increase of 4.3 percent in the odds of raising to a mature safety climate level. For maintaining a mature safety level, one additional point on Improvement, Patient-Centered and Commitment factors, was associated with an increase of 4.1, 6.2 and 3.7 percent, respectively. An explained variance (Nagelkerke R^2^) of 5.3 percent and 15.8 percent indicates that developments in safety climate might be explained by explanatory variables not included in our logistic model.

**Table 6:** Hospital unit work environment factors associated with the unit-level mature safety climate score.

Factor	Raising safety climate to a mature level (n = 440)* OR(95% CI)	Maintaining mature safety climate level (n = 530)* OR(95% CI)
Nagelkerke R^2^ (variance explained)	0.053	0.158
Improvement	1.043 (1.019, 1.068)	1.041 (1.007, 1.077)
Patient-Centered		1.062 (1.021, 1.105)
Commitment		1.037 (1.009, 1.066)

Factors significant in at least one of the models are presented.

* Adjusted for unit size.

## Discussion

The major findings of this study are the significant associations of organizational factors measured in the work environment survey and a change in the unit’s safety climate scores. The most prominent change factors associated with higher and mature safety climates were Improvement, Patient-Centered, Quality, and Commitment. These factors highlight the key organizational activities that ensure patient safety. The Improvement factor was significantly associated with all three study outcomes and displayed both the culture of reporting adverse events and the emotional characteristics of the unit environment where staff feel safe to speak up and "stop-the-line" if hazards are identified without fear of negative sanctions against them [44–46]. It could be argued that the Improvement factor is just one reflection of a safety climate: that is, perceived physiological safety and incident reporting is as likely to shape the safety climate as the safety climate supports staff attitudes [47]. However, McFadden et al. found that the patient safety climate and quality improvement were not interchangeable, but act in concert, and together can produce greater combined benefits [48]. We define quality in our survey based on the items teamwork and efficiency. It is widely recognized in the patient safety literature that teamwork and team performance are important in providing safe patient care [4,49]. A review by Manser [50] found that teamwork including coordination, communication, and leadership, are crucial to assuring patient safety. This finding suggests that strong unit networks and management resources for change are needed to create the important conditions for developing and nurturing a positive safety climate.

Patient preferences and views are essential sources for system co-design by making patient participation and agency a significant driver to attain better patient outcomes [51–54]. Patient-centered care calls for leadership styles that value patient contributions and encourage co-participation in decision-making [50,55]. There are multiple barriers to patient involvement, but engaged and involved employees are more likely to involve patients in a meaningful manner [56]. Organizational commitment may indicate a willingness to engage and make extra efforts to keep a work environment safe. Staff that perceive their work environment as supportive of their clinical practice, in which their views were valued, and the care improvement is the norm, are more likely to recommend their workplace to colleagues and patients [57]. The loyalty commitment that encourages staff to stay in their roles, and do their best may also affect patient safety outcomes.

Our analyses suggests that organizational targeted strategies to raise the safety climate to a mature safety level should be slightly differentiated from strategies aimed at maintaining a mature climate. We found that leadership efforts related to the Improvement factor are a key initiative for lifting a hospital unit to a mature climate level where more than 60% of the staff respond positively to the survey items. To maintain an established mature safety climate over time, the factors of Patient-Centered and Commitment are significant. A cautious interpretation could be that a safety climate is enabled when management is demonstrably focusing on quality and patient needs. However, to maintain a mature safety climate, the hospital management must go further, and create a nurturing and entrusting organizational setting that supports the staff to speak up when care is unsafe, and the staff feel committed, loyal, and actively involved in their unit’s improvement efforts.

This study has several limitations and must be interpreted in the context of its design. First, the staff survey measures the staff perception of their work environment and safety climate. We did not observe the actual unit work environment or culture, nor did we have objective clinical quality measures. Based on previous research we studied the safety climate at the hospital unit level as the variation in safety climate is more likely masked when aggregated to a hospital level [58,59]. We are aware, however, that not only the characteristics of each unit, but the overall organizational culture also influences the unit culture [60]. Moreover, hospitals represent a cultural mosaic consisting of several subcultures with varying values and attitudes not captured in this study [61]. Second, we did not include all the factors that could affect our results. Success and failures in developing an optimal patient safety climate in hospital units may depend on effectiveness of local leadership efforts to customize strategize at each hospital unit. Third, the study measured change in safety climate over time. We cannot rule out that the observed changes in the climate scores were due to unforeseen factors other than the ones measured. These limitations invite a more detailed analysis of factors affecting hospitals’ safety climate and unique unit characteristics over time and under variable environmental factors.

The study is susceptible to response bias. We used the longitudinal study design to assess staff perception of their work environment and safety climate in the same 970 hospital units over time. Our response rate compares favorably to response rates in other studies [62]. We are well aware that hospital staff might answer the survey questions untruthfully or misleadingly, for example, if they feel pressure to give socially acceptable answers or due to their fears of speaking up. These influences might include insecurity about the survey response anonymity, and the responders’ mood or cultural features. However, aggregating individual questionnaire responses across a unit lessens the effect of idiosyncratic or individual attitudes [63]. Finally, our study reflects the context and distinct constraints of the Norwegian healthcare system, which might differ from other healthcare systems and limit its generalizability. Norwegian employees generally perceive their work environment as more positive than staff in other countries [64]. Norwegian work life is highly regulated to secure staff’s physical and psychological wellbeing and national efforts such as monitoring staff perception on their work environment and safety climate are implemented in all Norwegian hospitals. Still variation was identified between the clinical units in our study, indicating the potential to improve the culture even where staff perceive their general work conditions as positive. We believe that our study’s results have relevance for the population as a whole and have external generalizability to other countries as the study dataset stems from a large and diverse representative sample of hospital units across South Eastern Norway.

## Conclusions

Our findings have important implications for hospital management practices. We demonstrated that the work environment characteristics were associated with significant changes in raising and maintaining a safety climate—essential for delivering safe and reliable care. Creating a hospital work environment where staff physical and psychological safety are a priority is key to an effective patient safety improvement strategy.

We believe that safety culture efforts should not be restricted to inspiring staff to reduce risks to their patients but should also include genuine staff buy-in and support of improvement efforts by hospital management to improve the usability and support for robust occupational environments.

## Supporting information

S1 STROBE checklist STROBE statement—Checklist of items that should be included in reports of observational studies. (DOCX)

S1 File (XLSX)

S2 File (DOCX)
