# Supplementary material for: Hospital work environments affect the patient safety climate: A longitudinal follow-up using a logistic regression analysis model
Source: PLoS One. 2021 Oct 15;16(10):e0258471. doi: 10.1371/journal.pone.0258471 (PMC8519418; doi:10.1371/journal.pone.0258471)
Supplement: S1 STROBE checklist — (DOCX) [file pone.0258471.s001.docx]

**STROBE Statement—checklist of items that should be included in reports of observational studies**

|  | Item No. | Recommendation | Page  No. | Relevant text from manuscript |
| --- | --- | --- | --- | --- |
| **Title and abstract** | 1 | (*a*) Indicate the study’s design with a commonly used term in the title or the abstract | Page 1 | Hospital work environment affects patient safety climate: A longitudinal sollow-up Study |
|  |  | (*b*) Provide in the abstract an informative and balanced summary of what was done and what was found | Page 2 |  |
| Introduction | | | |  |
| Background/rationale | 2 | Explain the scientific background and rationale for the investigation being reported | Page 3 |  |
| Objectives | 3 | State specific objectives, including any prespecified hypotheses | Page 5 | **Despite the emphasis on safety culture as an important strategy to patient safety we do not fully understand what factors might explain variation in culture. A relevant hypothesis might be that the work environment is related to how patient safety is handled in care giving unit. An opportunity to explore this hypothesis is to study the relation between staff perception on work environment and safety climate. This study aims to explore the association between work environment characteristics and the development in safety climate.** |
| Methods | | | |  |
| Study design | 4 | Present key elements of study design early in the paper | Page 6 | **This study was conducted with a longitudinal prospective design, combining data from the annual Work Environment Survey (WES) and safety climate data from the Norwegian Safety Attitude Questionnaire (SAQ).** |
| Setting | 5 | Describe the setting, locations, and relevant dates, including periods of recruitment, exposure, follow-up, and data collection | Page 6-10 | **970 clinical units in twenty one hospitals in South Eastern Norway. Survey data collected 2011, 2012 and 2014.** |
| Participants | 6 | (*a*) *Cohort study*—Give the eligibility criteria, and the sources and methods of selection of participants. Describe methods of follow-up  *Case-control study*—Give the eligibility criteria, and the sources and methods of case ascertainment and control selection. Give the rationale for the choice of cases and controls  *Cross-sectional study*—Give the eligibility criteria, and the sources and methods of selection of participants | Page 6 | **Hospital staff with more than three months or 30 % employment were included.** **The sample for this study was retrieved from clinical units participating in all three surveys with more than five responders in each unit and where no major reorganization had taken place between 2011 and 2014.** |
|  |  | (*b*) *Cohort study*—For matched studies, give matching criteria and number of exposed and unexposed  *Case-control study*—For matched studies, give matching criteria and the number of controls per case | NA |  |
| Variables | 7 | Clearly define all outcomes, exposures, predictors, potential confounders, and effect modifiers. Give diagnostic criteria, if applicable | Page 11 | **The primary outcome in the study was patient safety climate. We studied three specific outcomes associated with the development of a safety climate:**   1. **Change in safety climate score (2012 – 2014).** 2. **Raising safety climate to a mature level (>60% of staff scores 75 or higher).** 3. **Maintaining a mature safety climate over time** |
| Data sources/ measurement | 8* | For each variable of interest, give sources of data and details of methods of assessment (measurement). Describe comparability of assessment methods if there is more than one group | Page 6 | **The mean staff work environment scores were made available from the HSO. The data are assessed annually through the staff survey. The hospital unit safety climate score were made available by the National Patient Safety Campaign. Also collected through the patient safety survey conducted among staff in HSO.** |
| Bias | 9 | Describe any efforts to address potential sources of bias | Page 12 | **The analysis was adjusted for unit size.**  **In order to adjust for the potential for improvement, and to prevent regression to the mean acting as a confounder, the scores from SAQ_2012_ were included in the models where appropriate.** |
| Study size | 10 | Explain how the study size was arrived at | Page 6 | **All elegiable clinical units were included.** |

Continued on next page

| Quantitative variables | 11 | Explain how quantitative variables were handled in the analyses. If applicable, describe which groupings were chosen and why | Page 11 |  |
| --- | --- | --- | --- | --- |
| Statistical methods | 12 | (*a*) Describe all statistical methods, including those used to control for confounding | Page11-12 |  |
|  |  | (*b*) Describe any methods used to examine subgroups and interactions | NA |  |
|  |  | (*c*) Explain how missing data were addressed | Page 7 | **All statements had a “Not applicable” answer option. Answers of “non-applicable” were treated as missing data in the analysis.** |
|  |  | (*d*) *Cohort study*—If applicable, explain how loss to follow-up was addressed  *Case-control study*—If applicable, explain how matching of cases and controls was addressed  *Cross-sectional study*—If applicable, describe analytical methods taking account of sampling strategy |  |  |
|  |  | (*e*) Describe any sensitivity analyses | NA |  |
| Results | | | | |
| Participants | 13* | (a) Report numbers of individuals at each stage of study—eg numbers potentially eligible, examined for eligibility, confirmed eligible, included in the study, completing follow-up, and analysed | Page 12 | **Please see Tabel 3 in manuscript** |
|  |  | (b) Give reasons for non-participation at each stage | Page 7 | **Answers of non-applicability were treated as missing in the analysis.** |
|  |  | (c) Consider use of a flow diagram | NA |  |
| Descriptive data | 14* | (a) Give characteristics of study participants (eg demographic, clinical, social) and information on exposures and potential confounders | Page 6 |  |
|  |  | (b) Indicate number of participants with missing data for each vari able of interest | NA |  |
|  |  | (c) *Cohort study*—Summarise follow-up time (eg, average and total amount) | Page 6 |  |
| Outcome data | 15* | *Cohort study*—Report numbers of outcome events or summary measures over time | Page 13 | **Please see Fig.1 in manuscript** |
|  |  | *Case-control study—*Report numbers in each exposure category, or summary measures of exposure | *NA* |  |
|  |  | *Cross-sectional study—*Report numbers of outcome events or summary measures | NA |  |
| Main results | 16 | (*a*) Give unadjusted estimates and, if applicable, confounder-adjusted estimates and their precision (eg, 95% confidence interval). Make clear which confounders were adjusted for and why they were included | Page 13-15 | **Adjusted for SAQ_2012_ and unit size. Data was adjusted for unit size as larger units significantly reported lower WES scores than smaller units.** |
|  |  | (*b*) Report category boundaries when continuous variables were categorized | NA |  |
|  |  | (*c*) If relevant, consider translating estimates of relative risk into absolute risk for a meaningful time period | NA |  |

Continued on next page

| Other analyses | 17 | Report other analyses done—eg analyses of subgroups and interactions, and sensitivity analyses | NA |  |
| --- | --- | --- | --- | --- |
| Discussion | | | | |
| Key results | 18 | Summarise key results with reference to study objectives | Page 13-15 |  |
| Limitations | 19 | Discuss limitations of the study, taking into account sources of potential bias or imprecision. Discuss both direction and magnitude of any potential bias | Page 18-19 |  |
| Interpretation | 20 | Give a cautious overall interpretation of results considering objectives, limitations, multiplicity of analyses, results from similar studies, and other relevant evidence | Page 17 | **A cautious interpretation could be that a safety climate is enabled when management is demonstrably focusing on quality and patient needs. However, to maintain a mature safety climate, the hospital management must go further, and create a nurturing and entrusting organizational setting that supports the staff to speak up when care is unsafe, and the staff feel committed, loyal, and actively involved in their unit’s improvement efforts.** |
| Generalisability | 21 | Discuss the generalisability (external validity) of the study results | Page 19 | **The results might carry relevance for the population as a whole and have external generalizability to other countries because it stems from a large and diverse sample of hospital units** |
| Other information | |  | | |
| Funding | 22 | Give the source of funding and the role of the funders for the present study and, if applicable, for the original study on which the present article is based | NA |  |

*Give information separately for cases and controls in case-control studies and, if applicable, for exposed and unexposed groups in cohort and cross-sectional studies.

**Note:** An Explanation and Elaboration article discusses each checklist item and gives methodological background and published examples of transparent reporting. The STROBE checklist is best used in conjunction with this article (freely available on the Web sites of PLoS Medicine at http://www.plosmedicine.org/, Annals of Internal Medicine at http://www.annals.org/, and Epidemiology at http://www.epidem.com/). Information on the STROBE Initiative is available at www.strobe-statement.org.
