# Supplementary material for: Hospital work environments affect the patient safety climate: A longitudinal follow-up using a logistic regression analysis model
Source: PLoS One. 2021 Oct 15;16(10):e0258471. doi: 10.1371/journal.pone.0258471 (PMC8519418; doi:10.1371/journal.pone.0258471)
Supplement: S2 File — (DOCX) [file pone.0258471.s003.docx]

Medarbeiderundersøkelsen (MU)/**Work Environment Survey (WES)**

1. FORBEDRINGER **(IMPROVEMENT)**

1. I min enhet er vi flinke til å melde og følge opp avvik **(In my unit, we do well in reporting and follow up on adverse events)**

2. I vår enhet er det trygt å varsle om kritikkverdige forhold **(It is safe to report adverse events in my/this unit)**

3. Vi diskuterer åpent de feil og hendelser som oppstår for å lære av dem **(We openly discuss adverse events and learn from them)**

4. Vi oppmuntrer hverandre til å tenke ut måter å gjøre tingene bedre i min enhet **(In this unit, we encourage each other to think of ways to do things better)**

2. KVALITET **(QUALITY)**

1. I min enhet samarbeider forskjellige yrkesgrupper godt **(In my unit different professions collaborate well)**

2. I min enhet arbeider vi effektivt **(We work efficiently in my unit)**

3. I min enhet ivaretas høy faglig kvalitet **(In my unit high quality is maintained)**

3. TRYGGHET **(PATIENT-CENTERED)**

1. I min enhet lytter vi til pasientenes/servicemottakernes synspunkter **(In my unit, we listen to the views of patients/clients)**

2. I min enhet er vi lett tilgjengelige for pasienter/servicemottakere **(In my unit, we are available to patients/clients)**

3. I min enhet gir vi tilstrekkelig informasjon til pasienter/servicemottakere **(In my unit, sufficient information is given to patients/clients)**

4. RESPEKT **(RESPECT)**

1. I min enhet tar vi hensyn til pasientens/servicemottakerens livssyn og kulturelle bakgrunn **(In my unit, we respect patients’/clients’ cultural background and religion)**

2. I min enhet overholder vi de avtaler som blir gjort **(In my unit, we ensure that we keep made appointments)**

3. I min enhet blir ting sagt på en tydelig og forståelig måte **(In my unit, we communicate clearly and in an understandable way)**

5. MOTIVASJON **(MOTIVATION)**

1. Er arbeidet ditt utfordrende på en positiv måte? **(Is your work challenging in a positive way)**

2. Arbeidsoppgavene mine engasjerer meg **(My work tasks motivate me)**

3. Jobben er så interessant at den i seg selv er sterkt motiverende **(The work is so interesting in itself that it is strongly motivating)**

6. ARBEIDSGLEDE **(ENGAGEMENT)**

1. Gleder du deg til å gå på jobben? **(Do you look forward to go to work)**

2. Hvor ofte fører misnøye med jobben til at du ønsker å bytte arbeidsgiver? **(How often does dissatisfaction with your work make you want to change employer)**

3. Hvor fornøyd er du samlet sett med den jobben du har nå? **(Overall, how satisfied are you with the work you do now)**

7. TILHØRIGHET **(COMMITMENT)**

1. Jeg sier til mine venner at dette er en god arbeidsplass å jobbe på **(To my friends, I praise this organization as a great place to work)**

2. Denne arbeidsplassen inspirerer meg virkelig til å yte mitt beste **(This organization inspires me to give my very best job performance)**

3. Jeg er stolt av min arbeidsplass **(I am proud of my workplace)**

8. FAGLIG UTVIKLING **(PERSONAL DEVELOPEMENT)**

1. Får utvikle meg faglig gjennom jobben **(I can develop professionally through my work)**

2. Får tilstrekkelig undervisning og veiledning til å kunne gjøre en god jobb **(I get sufficient training and advice to do a good job)**

3. Legges det til rette for at du kan få utvikle dine ferdigheter **(Is your work organized in a way that lets you improve your capacities)**

4. Får du konstruktive tilbakemeldinger på arbeidet du utfører **(Do you get feedback about the quality of the work you do)**

9. MEDVIRKNING **(EMPOWERMENT)**

1. Oppmuntres du til å delta i viktige avgjørelser? **(Are you encouraged to participate in decision making)**

2. Oppmuntres du til å si ifra når du har en annen mening? **(Are you encouraged to speak up when you have a different opinion)**

10. ROLLEKLARHET **(ROLE EXPECTATION)**

1. Vet du hva som er ditt ansvarsområde? **(Do you know what your responsibilities are)**

2. Vet du nøyaktig hva som forventes av deg i jobben? **(Do you know what is expected of you at work)**

11. SOSIALT SAMSPILL **(SOCIAL CLIMATE)**

1. Er det sosiale klimaet i din enhet preget av medansvar og lagånd **(Is the social climate in your unit characterized by a team spirit)**

2. Om du trenger det, kan du få støtte og hjelp i ditt arbeid fra dine arbeidskolleger? **(If needed, can you get support and help from your coworkers)**

3. Opplever du at samarbeidet i enheten fungerer godt? **(Do you perceive good collaboration in your unit)**

12. KONFLIKTER **(CONFLICT)**

1. Har du lagt merke til om noen er blitt utsatt for mobbing eller trakassering i din enhet i løpet av de siste seks måneder? **(Have you observed anyone being harassed or bullied at your workplace during the last six months)**

2. Har du lagt merke til forstyrrende konflikter i din enhet? **(Have you noticed disruptive conflicts in your unit)**

3. Når konflikter oppstår i din enhet, blir de håndtert på en god måte? **(When conflicts occur, are they handled in a professional manner)**

13. ARBEIDSBELASTNING **(WORKLOAD)**

1. Er den fysiske arbeidsbelastningen for stor i arbeidet ditt? **(Is the physical load of your work too heavy)**

2. Er arbeidstempoet ditt belastende? **(Is your work pace challenging)**

3. Er arbeidsmengden din belastende? **(Is your workload challenging)**

4. Må du utføre arbeidsoppgaver du opplever at du ikke behersker? **(Do you perform work tasks for which you need more training)**

14. EGENKONTROLL **(AUTONOMY)**

1. Kan du påvirke mengden arbeid som blir tildelt deg? **(Can you influence the amount of work assigned to you)**

2. Kan du selv bestemme ditt arbeidstempo? **(Can you set your own work pace)**

15. ROLLEKONFLIKT **(ROLE CONFLICTS)**

1. Må du gjøre ting som du mener burde vært gjort annerledes? **(Do you have to perform procedures which you feel should be done differently)**

2. Får du oppgaver uten tilstrekkelig hjelpemidler og ressurser til å fullføre dem? **(Are you given assignments without adequate resources to complete them)**

3. Mottar du motstridende forespørsler fra to eller flere personer? **(Do you receive incompatible requests)**

16. JOBBRELATERT SYKEFRAVÆR **(SICK LEAVE)**

1. Jobbrelaterte forhold har vært medvirkende til sykefravær jeg har hatt (de siste 12 måneder) **(Issues at work have contributed to my sick leaves during the last 12 months)**

17. OPPLEVD LEDERADFERD **(LEADERSHIP)**

1. Min nærmeste leder er tilgjengelig for meg når jeg har behov for det **(My immediate superior is available to me when I need it)**

2. Min nærmeste leder er flink til å informere om det som skjer i vår virksomhet **(My immediate superior does an excellent job of giving us information about what goes on in our organization)**

3. Min nærmeste leder stiller tydelige krav til mine arbeidsprestasjoner **(My immediate superior makes clear performance demands)**

4. Min nærmeste leder følger opp det vi blir enige om **(My immediate superior adheres to what we have agreed upon)**

5. Dersom jeg ble utsatt for vold/trusler på jobb, ville jeg få god oppfølging fra nærmeste leder **(If I were subjected to violence or threats, I could count on the support of my immediate superior)**

6. Dersom jeg ble syk over en lengre periode, ville jeg få god oppfølging av nærmeste leder **(If I were sick for a more extended period, I could count on the support of my immediate superior)**

18. PASIENTSIKKERHETSKULTUR **(PATIENT SAFETY CULTURE)**

1. Jeg ville føle meg trygg hvis jeg var pasient her **(I would feel safe if I was a patient here)**

2. Her blir medisinske feil (behandlingsrelaterte forhold som gir/kunne gitt negativt utfall for pasient) håndtert riktig **(Adverse medical events are appropriately handled here)**
